# Effect of sulfonamide pollution on the growth of manure management candidate *Hermetia illucens*

**DOI:** 10.1371/journal.pone.0216086

**Published:** 2019-05-08

**Authors:** Qiao Gao, Wenhui Deng, Zhenghui Gao, Mengya Li, Wen Liu, Xiaoping Wang, Fen Zhu

**Affiliations:** Hubei International Scientific and Technological Cooperation Base of Waste Conversion by Insects, Huazhong Agricultural University, Wuhan, China; Gifu University, JAPAN

## Abstract

Antibiotics are commonly used in livestock and poultry farming. Residual antibiotics in manure may lead to antibiotic pollution of soil, surface water, and groundwater through land application and run-off rainfall. The black soldier fly (BSF) *Hermetia illucens* is a good candidate for manure management. We evaluated the effect of sulfonamide pollution on the growth of *H*. *illucens*. Four treatments were considered with a sulfonamide content in the feed of 0 (control group), 0.1, 1, and 10 mg/kg. The control larvae were fed without sulfonamide. Survival and development status of the individuals were recorded daily. The weights of the fifth instar larvae, prepupae, and pupae were checked. Antioxidant enzyme activity was determined with the fifth instar larvae. The results showed that a low (0.1 and 1 mg/kg) concentration of sulfonamides had no effects on larval survival, pupation, and eclosion of BSFs. A high sulfonamide concentration of 10 mg/kg had a significant effect on the survival of larvae and pupae and on the body weight of larvae, prepupae and pupae. Peak of the cumulated pupation rate and eclosion rate in the sulfonamide treatment of 10 mg/kg was very low. Pupation and eclosion in this group peaked later than that of the control and low sulfonamide concentration treatment groups (0.1 mg/kg and 1 mg/kg). Larvae from the sulfonamides group showed lower antioxidase activities than that of the control. In sulfonamide groups, the activity of peroxidase and superoxide dismutase was reduced in a concentration-dependent manner. Sulfamonomethoxine, sulfamethoxazole, and sulfamethazine were not detected in the harvested prepupae. Only sulfadiazine was discovered in the sulfonamide treatments of 1 and 10 mg/kg. In conclusion, BSFs can tolerate certain concentrations of sulfonamide contamination.

## Introduction

With the rapid growth of the global population and the increasing demands for food from animal origin, industrial livestock and poultry production have expanded significantly in recent decades [1, 2]. The yield of livestock manure reached 2.121 billion tons in 2011, which will continue to increase rapidly to 2.875 billion tons by 2020 and 3.743 billion tons by 2030 [3, 4]. The total amount of pig manure discharge is the first in several livestock manure [3, 4]. Subsequently, the large amount of livestock manure has created serious environmental pollution. Commonly, manure is used as organic fertilizer directly or after simple processing, such as anaerobic digestion or composting. The mismanagement of manure wastes may lead to greenhouse gas emissions, hazardous substances contamination and disease transmission [5–8]. Moreover, residual veterinary antibiotics in manure can be another environmental pollution problem that cannot be ignored. In many countries, antibiotics are widely used in livestock and poultry farming as feed additives for the prevention and treatment of infectious diseases or for improving feed efficiency and growth rate at a relatively low dose [9]. However, antibiotics in animal feed cannot be absorbed completely, and some of them are excreted with feces [10]. These contaminated feces might lead to secondary antibiotic pollution to soil, surface water and groundwater through land application and run-off rainfall [10–12]. Some studies have indicated that antibiotics in the soil can be absorbed and cumulated by crops, especially vegetables such as cucumbers, lettuce, radish and tomatoes, and finally enter into the food chain [10, 13]. These antibiotics eventually lead to the development of antibiotic resistance, which has resulted in the reduction of therapeutic potential against human and animal pathogens [14].

Sulfonamides (SAs) are some of the most frequently used antibiotics in human and veterinary medicine [15]. It has been estimated that more than 20,000 tons of SAs have been introduced into the biosphere each year. Additionally, 50–75% of those SAs were used in veterinary medicine. Sulfamonomethoxine (SMM), sulfamethoxazole (SMZ), sulfamethazine (SM2), and sulfadiazine (SD) are the most frequently used veterinary medicines [16]. SAs can be easily found in rivers, surface water, soil and even in food due to the excessive use and high mobility of these antibiotics [13, 17, 18]. A wide range of SMZ concentrations spanning from 6 μg/L to 50 mg/L was detected [19–22]. Source water, drinking water, and distribution system (tap) water from 19 U.S. water utilities were analyzed for 51 compounds between 2006 and 2007. SMZ was included in the 11 most frequently detected compounds. The concentration of SMZ in source water was 12 ng/L [23]. A survey about antibiotics in drinking water in Austria showed that SMZ was detected in 10 of 200 samples collected in 2014. Five samples showed concentrations above the limit of quantification (2.5 ng/L) [24]. In Jiangsu province of China, SD and SMZ was include in 14 detected antibiotics in source water. The concentration was 1.2 to 3.2 ng/L for SD and 1.5–4.7 ng/L for SMZ [25]. Several studies have estimated the ecotoxicity of SAs. For example, Ahmed et al. (2015) reported that SAs in the soil could affect the growth of plants [13]. SMZ at the environmental exposure level could inhibit the growth of HEK293 human embryonic cells [26]. Anna et al. (2011) showed that SAs can seriously endanger nontarget organisms, such as green algae and duckweed, at environmentally relevant concentrations [27]. Most importantly, SA-induced antibiotic resistance of microbes could be potentially hazardous to human health [16].

Therefore, a more sustainable approach is needed to minimize waste and eliminate antibiotics at the same time. Traditional manure management, like composting, mainly focuses on waste minimization, but residual veterinary antibiotics remain in the residue. Recent studies have reported that some insects, such as house flies (*Musca domestica* L.), can attenuate antibiotics when vermicomposting animal manure [28]. In addition, because of the strong ability of these insects to reduce manure amount and the high content of protein and lipids in the resulting biomass, saprophagous insects such as house flies and black soldier flies have been widely used in manure management [29–31]. Therefore, these insects may be good bioreactors for antibiotic-contaminated manure waste disposal. The insects can not only transform the manure mass into animal protein and lipids but also attenuate the contained antibiotics [32–34].

Black soldier fly (BSF) is a good candidate for manure management. The larvae have a high ability to consume a wide range of organic waste [35] and generate biomass used as additive and substitute for livestock, poultry and fish feed [36–38]. The rich lipid content of the biomass generated makes BSF a potential raw material source for biodiesel production [39–41].

Trimethoprim is known to enhance the activity of sulfonamides on a wide variety of microorganisms [42]. Half-life of trimethoprim was shorter in the BSF compost (< 10% of control) and no bioaccumulation was detected in the larvae [43]. Tetracycline (TC) was also a representative antibiotic [44]. BSF could degrade 97% tetracycline within 12 days [45]. However, effects of sulfonamide on BSF and sulfonamide degradation by BSF remained unclear. The main objective of this research was to evaluate the effect of sulfonamide-contaminated feed on the growth and development of BSF.

## Materials and methods

### Source of insects

BSF were provided by the lab of Hubei International Scientific and Technological Cooperation Base for Waste Conversion by Insects, Huazhong Agricultural University, China. According to the literature [46], the eggs were kept at 27 ± 1°C and 60 ± 5 RH for hatching. Neonatal larvae were fed with wet wheat bran (50 g wheat bran: 100 mL H_2_O) for 4 days at 27 ± 1°C before subsequent experimental treatments.

### Preparation of SA solution

Chromatographically pure compounds (> 98%) of sulfamonomethoxine (SMM), sulfamethoxazole (SMZ), sulfamethazine (SM2), and sulfadiazine (SD) were purchased from Shanghai Yuanye Bio-Technology Co., Ltd, China. Equal amounts of SD, SM2, SMM, and SMZ (0.02 g) were dissolved in 10 mL methyl alcohol. After that, this solution was diluted to 5 mg/L, 0.5 mg/L, and 0.05 mg/L solution.

### Experimental design

Dry wheat bran was moistened with experimental SA solution in a ratio of 50 g wheat bran: 100 mL solution. The final SA content in the feed was 0.1 mg/kg, 1 mg/kg, and 10 mg/kg. The feed was placed in a plastic box (10 cm in diameter, 8 cm high). In each container, 100 4-d-old larvae were inoculated. All containers were then covered with a cloth fixed by rubber bands to prevent the larvae from escaping. The control larvae were fed with wheat bran moistened by ddH_2_O. Each treatment had three replicates.

The development status of the larvae were checked and the numbers of larvae and prepupae were counted and recorded daily. When more than 20% of the individuals reached the prepupal stage, the number of larvae and prepupae were recorded. Prepupal dynamics were observed until the pupation stage. Once the pupae began to appear, the number of pupae was counted every day. The number of adults was recorded every day when emergence occurred. The weights of the fifth instar larvae, prepupae, and pupae were checked. Groups of 20 individuals were washed with ddH_2_O and dried with absorbent paper before being weighed.

### Determination of the antioxidant enzyme activity

One hundred 4-d-old larvae were fed with 0.1, 1, or 10 mg/kg SA-containing wheat bran. The control larvae were fed with wheat bran without added SAs. Each treatment had three replicates. The fifth instar larvae were sampled for determination of enzyme activity. In each sampling, 15 larvae per replicate were washed with ddH_2_O three times, dried with absorbent paper, and then weighed before being stored at -80 °C. Determination of the protein content and enzyme activity was performed according to the protocol of the assay kit supplied by the manufacturer. The article numbers of the assay kit for detection of total protein, catalase, peroxidase, superoxide dismutase, and total antioxidant capacity were A045-2, A007-1, A084-1, A001-1, and A015, respectively. All the assay kits were purchased from Nanjing Jiancheng Institute of Biological Engineering, China.

### SA content determination

Ten prepupae were randomly selected from each replicate. The extracting agent was the mixture of methanol and 0.1% acetic acid (30:70, V/V). Ten prepupae with 2 mL extractant were homogenated in a grinding machine (JXFSTPRP-24, Shanghai Jing Xin Industrial Development Co., Ltd, China). The crude extraction was centrifuged at 4 °C and 4000 rpm for 10 min. The precipitate was homogenated again with above steps. The combined supernatant was filtered through a 0.45-μm membrane before injection into High Performance Liquid Chromatography (HPLC) system.

HPLC equipped with a VWD detector was used to determine the SA content of the larvae. The optimal detection conditions were determined as follows: HPLC instrument fixed with Agilent Eclipse XDB C-18 column (4.6 × 150 mm, 5 μm), mobile phase methanol-0.1% acetic acid (30:70, V/V) pumped at a flow rate of 0.8 mL/min, and the operating temperature of the column was at 30 °C. The detection wavelength was 270 nm.

### Statistical analysis

Larval survival rate was calculated as the number of survived larvae divided by the number of the larvae in the beginning. Differences in mean value of developmental deviation (prepupae rate, pupation rate, eclosion rate, weight, duration, and activities of antioxidases) among different treatments were tested in SPSS software (IBM SPSS Statistics 19) using one-way ANOVA. Obtained data were analyzed by Duncan test. *P* < 0.05 was accepted as the level of significance for all analyses.

## Results

### Survival rate

**Fig 1:**
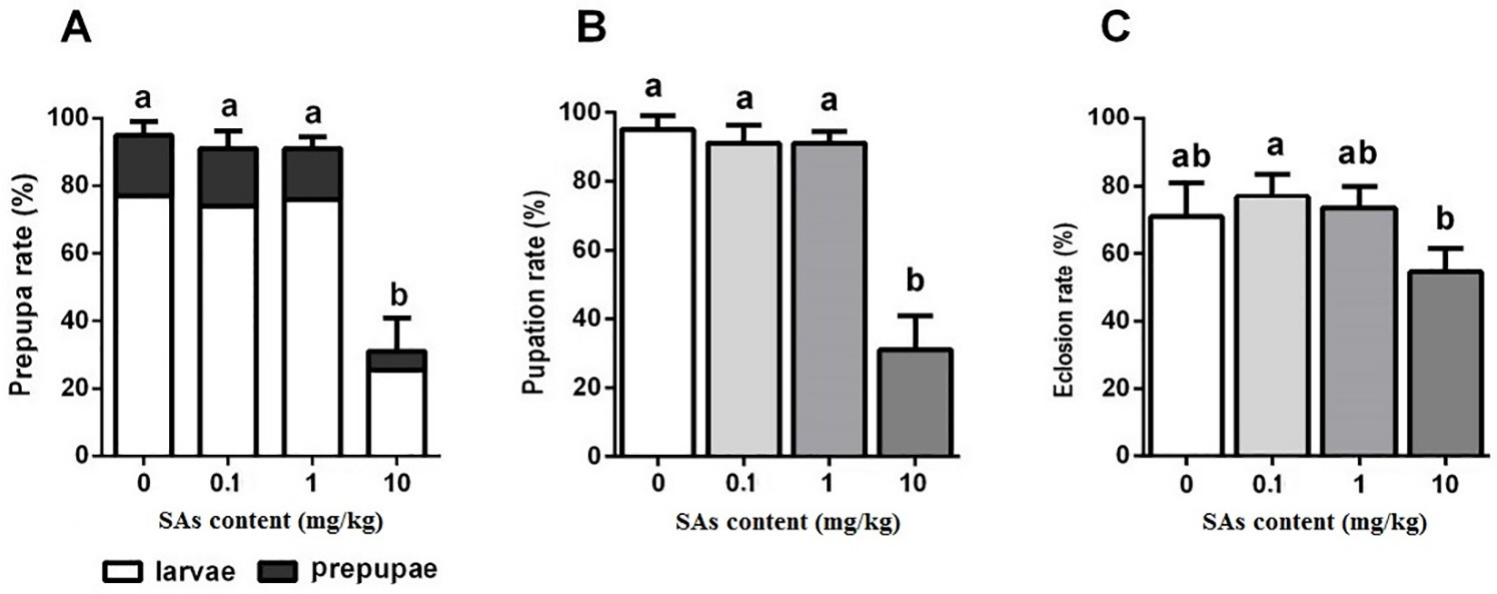
Effect of SAs-contained diet on the prepupae rate, pupation rate, and eclosion rate of the black soldier flies. Differences in mean value (n = 3) of different treatments were tested by one-way ANOVA and Duncan test. Different letter showed significant difference (P< 0.05).

When 20% of individuals reached the prepupal stage, no significant difference in larval survival rate was found between the treatment with low SA content (0.1 and 1 mg/kg) and the control. There was only 30% larval survival in the SA treatment of 10 mg/kg (Fig 1A). The pupation rate among the control and the SA treatments of 0.1 mg/kg and 1 mg/kg did not vary significantly. The SA treatment of 10 mg/kg made a significant difference in pupation rate (Fig 1B). Only 30% of individuals reached the pupal stage in the SA treatment of 10 mg/kg, which was significantly lower than that of the control and the SA treatments of 0.1 mg/kg and 1 mg/kg. Over 70% of the pupae emerged successfully in the control and in SA treatments of 0.1 mg/kg and 1 mg/kg. For the SA treatment of 10 mg/kg, the eclosion rate was approximately 55%, which was significantly lower than the rate in the other treatments (Fig 1C).

### Body weight

**Fig 2:**
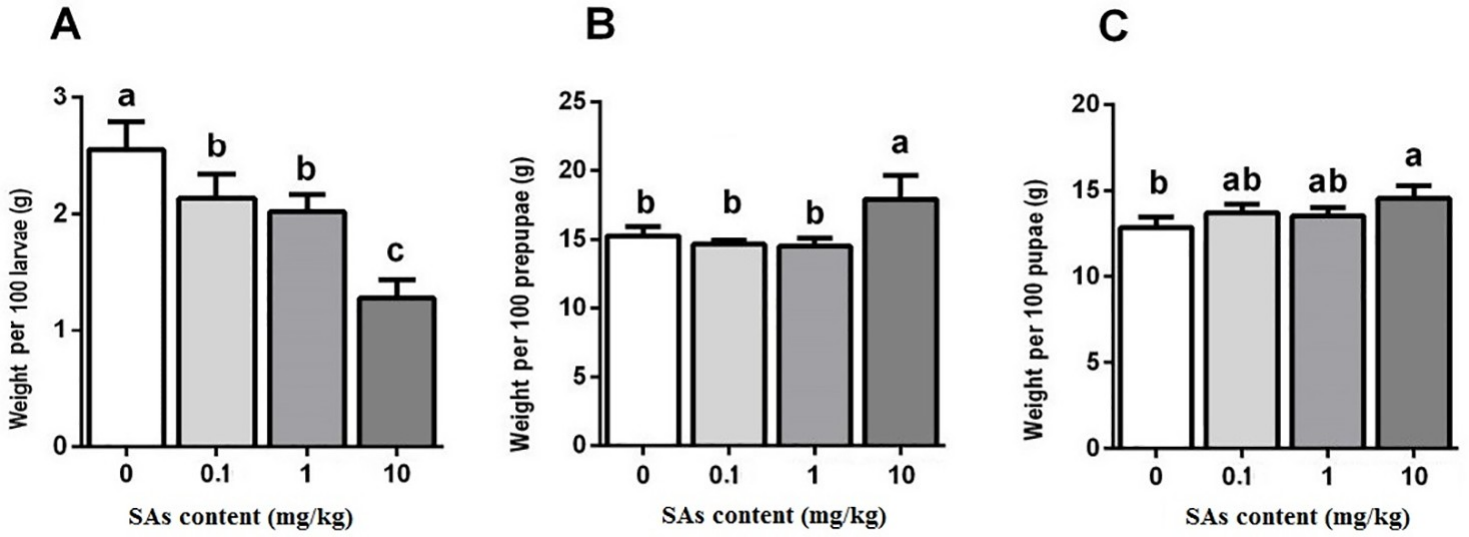
Effect of SAs-contained diet on individual weight of the black soldier flies. Differences in mean value (n = 3) of different treatments were tested by one-way ANOVA and Duncan test. Different letter showed significant difference (P<0.05).

When fed with the SA-containing diets, the body weight of the larvae was remarkably lower than that of the control (Fig 2A). With the increase in SA concentration, the weight of the larvae declined gradually. The lightest larvae were discovered in the SA treatment of 10 mg/kg, which was only 51.15% of the weight of the control larvae (Fig 2A). With the SA-containing diet in the larval stage, the body weight of the prepupae was not significantly affected by low SA levels (0.1 mg/kg and 1 mg/kg) but increased at the highest SA level (Fig 2B). Pupae weight was similar among the control and the SA treatments of 0.1 mg/kg and 1 mg/kg (Fig 2C). The number of larvae that successfully reached the prepupae and pupae stage in the SA treatment of 10 mg/kg was significantly greater than that of the control (Fig 2B and 2C).

### Duration of the larval stage and pupal stage

**Fig 3:**
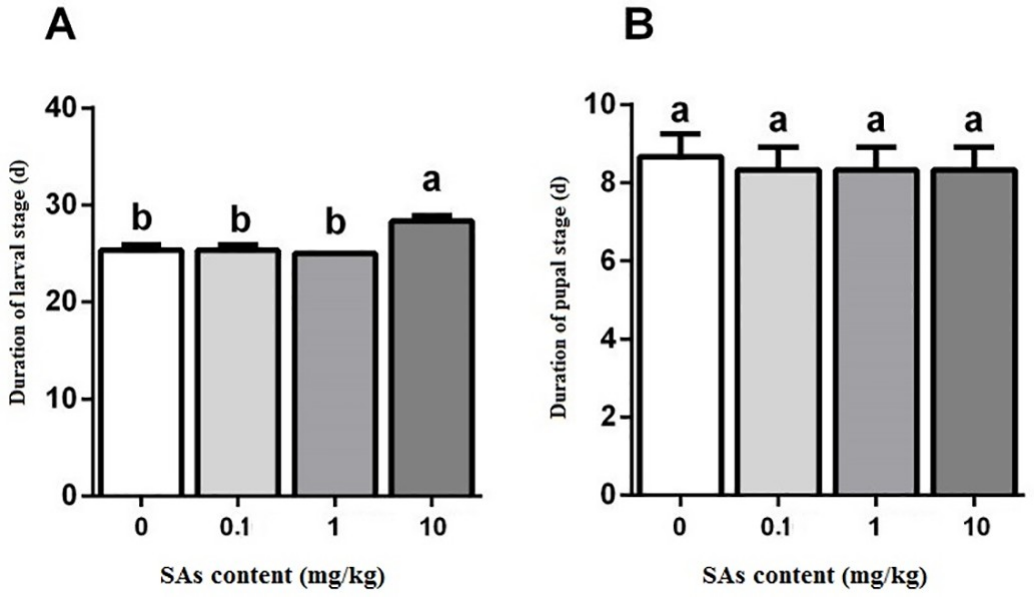
Effect of SAs-contained diet on the larval duration and pupal duration of the black soldier flies. Differences in mean value (n = 3) of different treatments were tested by one-way ANOVA and Duncan test. Different letter showed significant difference (P<0.05).

Low SA content (0.1 mg/kg and 1 mg/kg) in the fly diet did not show a noteworthy effect on larval stage duration when compared with the control group (Fig 3A). The larval stage duration of the SA treatment of 10 mg/kg was the longest, 3 days longer than that of the other. Experiencing SAs did not affect pupal development. The duration of the pupal stage among the control and the SA treatments of 0.1 mg/kg, 1 mg/kg, and 10 mg/kg did not differ significantly (Fig 3B).

### Pupation dynamics and eclosion dynamics

**Fig 4:**
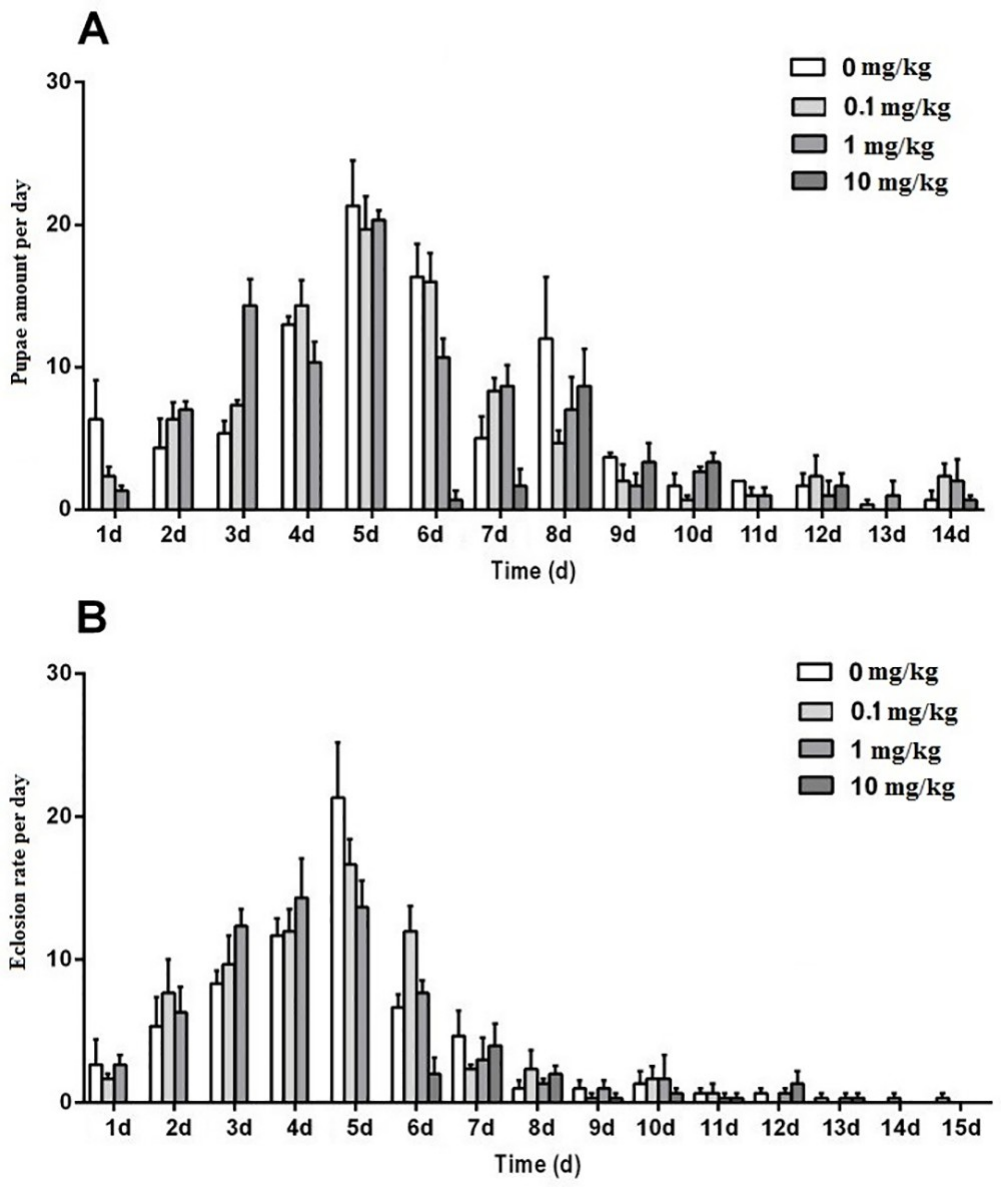
Effect of SAs-contained diet on pupation dynamics and eclosion dynamics of the black soldier flies when experienced SAs-contained diet in larval stage. The value was mean ± SD (n = 3).

The appearance of the first pupa in the control and the SA treatments of 0.1 mg/kg and 1 mg/kg was 5 days earlier than that in the SA treatment of 10 mg/kg. The peak time for pupation was delayed 3 days at the highest SA concentration when compared with the control and the low SA treatments (0.1 mg/kg and 1 mg/kg) (Fig 4A). On the peak pupation day, the cumulated pupation rates in the control and the SA treatments of 0.1 mg/kg and 1 mg/kg were 67%, 60%, and 63%, respectively. There was just a 12% cumulated pupation rate for the SA treatment of 10 mg/kg until the eighth day, which was only 14.28% of that in the control group (Fig 4A).

The average pupal stage was 9 days. The earlier an individual pupated, the earlier the adult emerged. The appearance of the adults in the SA treatment of 10 mg/kg was 5 days later than that of the other. The eclosion peak in the control and low SA treatment groups (0.1 mg/kg and 1 mg/kg) was on the fifth day, which was 2 days earlier than that of the SA treatment of 10 mg/kg (Fig 4B). On the peak eclosion day, the cumulated eclosion rates in the control and the SA treatments of 0.1 mg/kg and 1 mg/kg were 48%, 46%, and 47%, respectively. There was only an 8% cumulated eclosion rate in the SA treatment of 10 mg/kg on the peak eclosion day (Fig 4B).

### The activity of the antioxidant enzyme system

**Fig 5:**
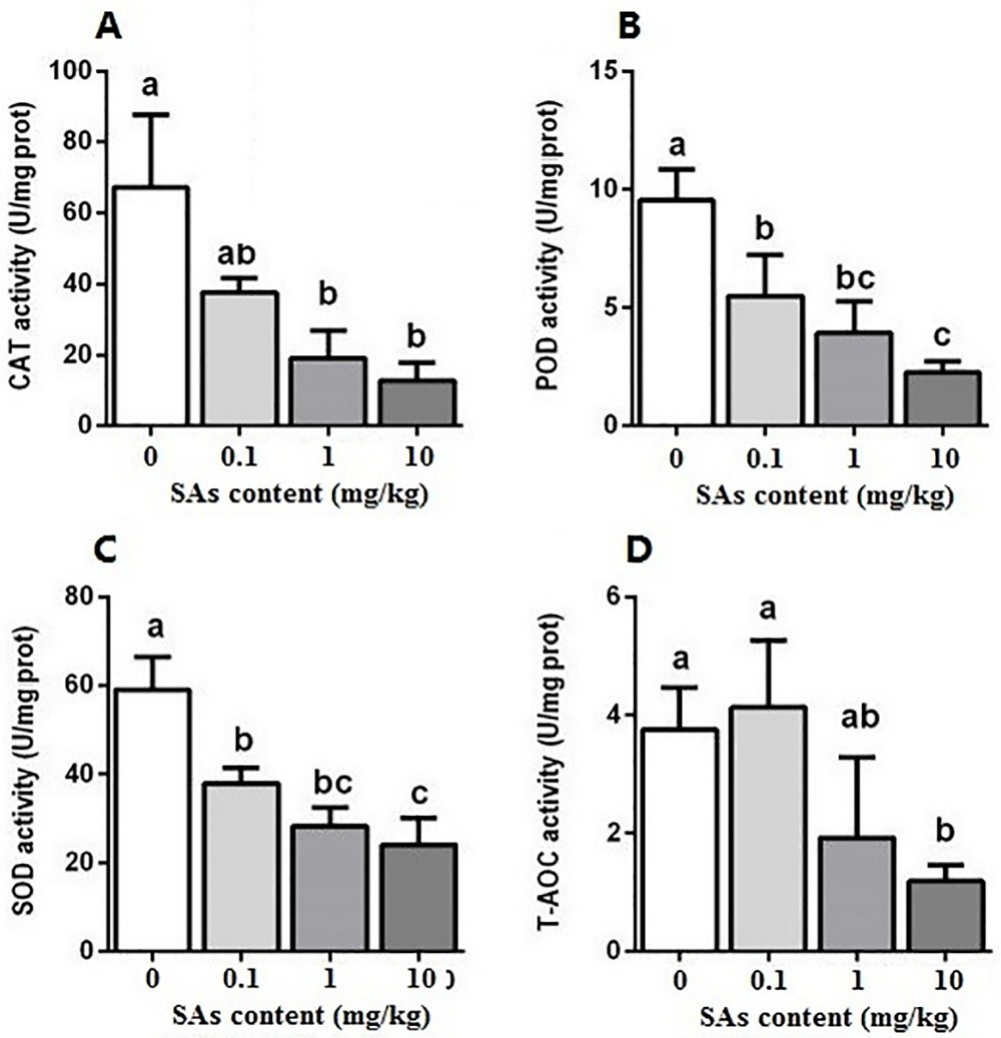
Effect of SAs-contained diet on activities of antioxidases in larvae of the black soldier flies. Differences in mean value (n = 3) of different treatments were tested by one-way ANOVA and Duncan test. Different letter showed significant difference (P<0.05).

The activities of total protein, catalase (CAT), peroxidase (POD), superoxide dismutase (SOD), and total antioxidant capacity (T-AOC) are shown in Fig 5. The activity of CAT in the larvae was reduced when SAs were added to the diet. The control larvae had the highest CAT activity, which was 3.51 times that in the SA treatment of 1 mg/kg and 5.25 times that in the SA treatment of 10 mg/kg. The concentration of SAs did not show a significant effect on the CAT activity of the larvae (Fig 5A).

SA treatment can greatly reduce the activity of POD in larvae in a concentration-dependent manner. The activity of POD in the larvae from the SA treatments of 0.1, 1, and 10 mg/kg sharply decreased to 57.57%, 41.3%, and 23.6%, respectively, compared to the control larvae. There was no sharp difference in POD activity of the larvae between the SA treatments of 0.1 and 1 mg/kg and between the SA treatments of 1 and 10 mg/kg (Fig 5B). The POD activity of the larvae from the SA treatment of 0.1 mg/kg was 2.44 times that in the SA treatment of 10 mg/kg.

The activity of SOD in the larvae greatly decreased with the increase of SA concentration. Larvae from the SA treatments had significantly lower SOD activity than that of the control larvae. The SOD activity of the larvae from the SA treatments of 0.1, 1, and 10 mg/kg was markedly reduced by 64.29%, 47.97%, and 40.76%, respectively, when compared to the control larvae. The highest SA treatment (10 mg/kg) caused larval SOD activity to decrease to 40.98% of the SA treatment (0.1 mg/kg). No remarkable difference was found in SOD activity of the larvae between SA treatments of 0.1 and 1 mg/kg and between SA treatments of 1 and 10 mg/kg (Fig 5C).

Changes in T-AOC activity in the larvae did not linearly depend on SA concentrations. There was no significant difference in the T-AOC activity of the larvae among the control and SA treatments of 0.1 and 1 mg/kg. Larvae from the SA treatment of 10 mg/kg had the lowest T-AOC activity (Fig 5D).

### SA content analysis

The SA content in the harvested prepupae is shown in Table 1. Only sulfadiazine (SD) was found in treatments of 1 and 10 mg/kg. Sulfamethazine (SM2), sulfamethoxazole (SMZ), and sulfamonomethoxine (SMM) were undetectable.

**Table 1:** SA content in the harvested prepupae.

Treatment (mg/kg)	Content (mg/ 100 prepupae)			
	SD	SM2	SMZ	SMM
0.1	/	/	/	/
1	0.4663 ± 0.0575	/	/	/
10	0.7814 ± 0.3255	/	/	/

Note: SD: sulfadiazine, SM2: sulfamethazine, SMZ: sulfamethoxazole, SMM: sulfamonomethoxine. /: undetectable. The value was mean ± SD, n = 3.

## Discussion

### SA exposure affects the growth parameters of the black soldier fly

In this research, SAs showed effects on the growth and survival of BSF. However, the impact extent depended on the developmental stages of BSF and doses of SAs. High level SAs treatment of 10 mg/kg had a remarkable effect on survival of larvae and pupae and on body weight of prepupae and pupae (Figs 1, 2 and 3). Low level SAs treatments (0.1 mg/kg and 1 mg/kg) did not show impact on larval survival, and pupation to BSF (Fig 1). In the literature about *Fennero penaeus chinensis*, the 24h-LC_50_ of sulfamethoxazole (SMZ) against the nauplii stage and zoea stage of larvae were 300 and 355 mg/L, respectively, and the 48h-LC_50_ were 232 and 172 mg/L, respectively [47]. This suggests that different organisms at different developmental stage can tolerate different concentrations of SAs.

Reproduction is another important indicator for understanding the potential ecological effects of some stressors on living organisms. Cumulated pupation rate and cumulated eclosion rate of BSF in SAs treatment of 10 mg/kg was very low when compared to other groups (Fig 4), which can lead to fewer adults and subsequently fewer offspring. Not only that, delayed pupation and eclosion may cause the adults miss the matting days, leading to the failure of reproduction.

### SA exposure affects the antioxidant enzyme system of the black soldier fly

To address the harmful effects of oxidative stress originating from xenobiotic exposure, living things are equipped with numerous defense mechanisms, including changes in antioxidant enzymes. Antioxidant enzymes such as SOD, CAT, and others can be used as biomarkers that suggest the beginning and the level of the responses to xenobiotic exposure [48]. In this study, SA treatment greatly reduced the activity of POD and SOD in BSF larvae in a concentration-dependent manner (Fig 5). BSF larvae from the SA group had lower antioxidase activities than that of the control group (Fig 5). This suggests that the ability of the antioxidase-based scavenging of free radicals was decreased in SA-treated larvae. However, catalase and glutathione S-transferase of *Daphnia magna* showed concentration-dependent increases caused by sulfathiazole exposure [38]. In the tubifex *Monopylephorus limosus*, SMZ could induce oxidative stress, and CAT played a key role in removing oxygen free radicals [49].

### SA attenuation by the black soldier fly

BSF larvae can attenuate some sulfonamides in feed substances. Sulfamethazine (SM2), sulfamethoxazole (SMZ), and sulfamonomethoxine (SMM) were undetectable in the harvested prepupae. Only sulfadiazine (SD) was found in the preupae at treatment levels of 1 and 10 mg/kg (Table 1). In swine manure bioconversion with housefly larvae (*Musca domestica*), nine antibiotics were drastically reduced, including tetracyclines, sulfonamides, and fluoroquinolones [28]. These results suggest that some insects can be used for antibiotic attenuation. Manure treatment with BSF could thus reduce the risk of spread of antibiotic in the environment when applying composted material to arable land. Furthermore, the harvested insects with high protein and lipid content have great implications for livestock and pet nutrition.

## Conclusion

By studying the effect of sulfonamide pollution on the growth of *H*. *illucens*, we found that BSF larvae have the ability to tolerate certain levels of sulfonamide contamination and may be used for sulfonamide attenuation, especially for sulfamonomethoxine, sulfamethoxazole, and sulfamethazine. This promising ability is encouraging, especially for reducing antibiotic residues in the environment. Level of SA contamination in rearing substrates less than 10 mg/kg is usable by BSF. However sulfadiazine (SD) is found in the preupae at treatment levels of 1 and 10 mg/kg. This suggests that some other ways should be found for SD attenuation.

## Supporting information

S1 Table DATA used to build graphs. (RAR)
